# Association between protein intake and lean body mass in a group of Masters Athletes

**DOI:** 10.1017/jns.2022.10

**Published:** 2022-04-27

**Authors:** Joseph R. Stanzione, Joseph I. Boullata, Michael L. Bruneau, Stella L. Volpe

**Affiliations:** 1 Worldwide Sport Nutritional Supplements Bohemia, Bohemia, NY, USA; 2 Hospital of the University of Pennsylvania, Philadelphia, PA, USA; 3 Department of Health Sciences, Drexel University, Philadelphia, PA, USA; 4 Department of Human Nutrition, Foods, and Exercise, Virginia Polytechnic Institute and State University, 295 West Campus Drive (MC 0430), 338 Wallace Hall, Blacksburg, VA, USA

**Keywords:** DXA, Lean body mass, Masters Athletes, Protein, LBM, lean body mass, US RDA, US Recommended Dietary Allowance

## Abstract

Recommendations for protein intake are based on total body weight; however, these recommendations do not consider lean body mass (LBM). The purpose of the present study was to identify the average protein intake in g/kg LBM in a group of healthy Masters Athletes (≥26 years of age, exercising ≥2 d/week). Data were obtained from a cross-sectional study. Body weight (kg), height (cm) and LBM via dual-energy X-ray absorptiometry were measured. Dietary intake was measured using a 2005 Block Food Frequency Questionnaire. The average energy intake, the percent energy from protein and the average protein intake in g/kg LBM were calculated. Differences between protein intake and the US Recommended Dietary Allowance (US RDA) (0⋅8 g/kg body weight) were determined. Alpha levels were set *a priori* to *P* < 0⋅05. A total of 176 participants (94 women, 82 men; 39 ± 11 years of age; body mass index: 24⋅6 ± 3⋅4 kg/m^2^) were analysed. The average energy intake, the percent protein energy and the average protein intake were 7996⋅9 ± 110⋅9 kilojoules (kJ)/d (1,910⋅4 ± 26⋅5 kcal), 15⋅5 ± 2⋅6 % and 1⋅43 ± 0⋅53 g/kg LBM, respectively. No differences existed between women and men for protein intake/kg LBM. Both sexes had significantly higher protein intakes than the US RDA (*P* < 0⋅001). We identified the average protein intake (g/kg LBM) in healthy Masters Athletes that may contribute to evolving perspectives on the determination of protein needs. The present study helps establish the relationship between protein intake and LBM so that we may further increase our accuracy when developing future protein recommendations.

## Introduction

Dietary protein is a crucial nutrient in the human diet that is essential for maintaining cellular function and body composition. Intake recommendations for dietary protein in healthy adults have been established using the estimated average requirement (EAR) to determine the United States Recommended Dietary Allowance (US RDA). Rather than providing absolute values, the dietary recommendation for protein intake is 0⋅80 gram per kilogram (g/kg) of total body weight; however, these recommendations do not provide disaggregated reference recommendations for adults by age or sex. Instead, the adult recommendations for dietary protein were based on meta-analyses of studies evaluating the daily protein intake needed to achieve zero-nitrogen balance as its endpoint^(1)^. Maintaining zero-nitrogen balance is an indirect marker for the maintenance of protein needs as a measurement of protein breakdown to protein synthesis. A zero-nitrogen balance, therefore, equates to an equilibrium of protein breakdown to synthesis, which may be integral to the establishment of protein needs; however, nitrogen balance fails to identify mechanisms for such processes and their implications in lean body mass (LBM). During times of energy balance, zero-nitrogen balance does not directly account for the maintenance of LBM, and therefore clinically, it has its limitations.

Limitations of the nitrogen balance studies conducted to date are well recognised, and incorporation of outcome measures for protein intake, such as maintaining or improving LBM, are needed to better reflect physiologic needs^(2–4)^. Although protein recommendations are proportionate to total body weight, recent evidence suggests that LBM may provide a more accurate and precise metric for dietary protein needs given the known relationship between dietary protein and body composition in the literature^(3–5)^.

Data exploring the relationship between LBM and protein intake are also limited and are often found in correlational studies with older adults living with or who are at-risk for sarcopenia^(6,7)^. Finally, research that has examined outcomes for effective protein intake have revealed a possible range between 0⋅83 g and 1⋅77 g/kg LBM, well above the US RDA using total body weight^(4,8)^. Given the large inter-individual variability in body composition, coupled with nitrogen balance as an intermediate marker that reflects body cell mass or its clinical proxy, LBM, it may, therefore, be more appropriate to consider LBM when analysing adult protein needs.

Based on the limited research available for LBM in relation to protein need, more data are needed to better understand the relationship between these two variables. Data capturing the relationship between protein intake and LBM are needed to elucidate protein needs that can be based on LBM as opposed to total body mass. The most representative sections of the population who are likely meeting their daily protein needs and nitrogen balance are healthy, weight stable adults. Previous researchers have elucidated that Masters Athletes have similar muscle characteristics, physiological responses to exercise and protein metabolism as young athletes, and are unlikely to have protein requirements that are different from their young contemporaries^(9)^. We, therefore, selected a cohort of Masters Athletes to better articulate the relationship between LBM and protein intake. In addition, there is a paucity of data on protein needs for Masters Athletes. The primary aims of the present study were to compare protein intake to the current guidelines as well as describe the average protein intake in g/kg of LBM in a group of healthy adult Masters Athletes as a first step to exploring the relationship between dietary protein intake and LBM. We hypothesised that dietary protein intake would be significantly different from current intake guidelines.

## Materials and methods

### Participants

Data were obtained from a cross-sectional study in a cohort of healthy Masters Athletes. Masters Athletes were defined as athletes, 26 years of age and older, who exercised at least 2 d/week. Our population represented a diverse group of sports, in which the age of Masters Athletes varied from as young as 21 years of age. Therefore, we used 26 years of age as the starting age for those who would be grouped as Masters Athletes. Participants were excluded if they were active smokers, pregnant women or diagnosed with an uncontrolled chronic disease. Recruitment was conducted using posted flyers around the Philadelphia area. The present study was conducted according to the guidelines laid down in the Declaration of Helsinki and all procedures involving human subjects/patients were approved by the Institutional Review Board at Drexel University (Approval No. 1304002037). Written and verbal informed consent was obtained from all subjects/patients.

### Anthropometric and body composition assessment

#### Body weight, height and body mass index

Participants were measured for body weight (kg) and height (cm), using a mechanical column scale and an attached stadiometer (Seca Hamburg, Germany), respectively. Body weight and height were measured twice, and the average of the measurements was computed and recorded as a quality control measure. Body mass index (BMI) was calculated for each participant using their weight in kg divided by height in metre squared (kg/m^2^).

#### Dual-energy X-ray absorptiometry

Body composition and LBM were measured using dual-energy X-ray absorptiometry (DXA; LunariDXA, General Electric Company, 2018). DXA is considered one of the most accurate methods for body composition assessment due to its demonstrated validity, reliability and precision^(10–13)^. Participants subjected to the DXA scan received a total body scan to assess LBM, which was interpreted in pounds. LBM was then converted from pounds to kg for analysis.

### Energy and protein intakes

To assess total energy and protein intakes, each participant completed a self-administered 2005 Block's Food Frequency Questionnaire (FFQ)^(14,15)^. The FFQ is a previously validated tool that produces data representative of yearly dietary consumption by asking questions about dietary habits and specific food consumption. The FFQ has been shown to accurately predict protein intake when compared to 4-d diet records^(14)^. Completed questionnaires were sent to NutritionQuest© (Berkeley, CA) for analysis and returned to the research team for interpretation and statistical analyses.

### Statistical analyses

Descriptive statistics (mean ± standard deviation) were used to determine the average energy intake, the percent energy intake from dietary protein and the average protein intake in g/kg of LBM. An independent samples *t*-test was used to determine whether differences existed in protein intake (g/kg LBM) between women and men. A one-sample *t*-test was also used to compare the average protein intake of participants (total sample, and by women and men) to the US RDA (0⋅8 g/kg body weight). A *post hoc* power analysis was applied with an effect size of 0⋅5 and with a sample size of 176, which resulted in a power of 0⋅99 for the total sample. All statistical procedures were performed with the Statistical Package for the Social Sciences (SPSS) version 24.0 with alpha levels set *a priori* to *P* < 0⋅05.

## Results

### Participant characteristics

A total of 176 Masters Athletes (94 women, 82 men) were included in our analyses (Table 1).

**Table 1.** Participant characteristics of the total sample

	Total sample (*n* 176)	Standard Deviation	Women (*n* 94)	Standard Deviation	Men (*n* 82)	Standard Deviation	*P* value
Age (years)	39	11	40	10.4	37⋅6	10.8	0⋅13
BMI (kg/m^2^)	24⋅6	3.4	24⋅2	3.7	25⋅1	3.1	0⋅13
Height (m)	1⋅71	0.10	1⋅65*	0.07*	1⋅80	0.07	<0⋅001
Weight (kg)	72⋅86	13.5	66⋅0*	11.0*	80⋅7	11.8	<0⋅001
Lean body mass (kg)	51⋅8	10.7	43⋅7*	5.0*	61⋅1	7.3	<0⋅001

BMI, body mass index; kg, kilograms; m, metres.

* Women's values are significantly lower than men's values.

**Fig. 1.**
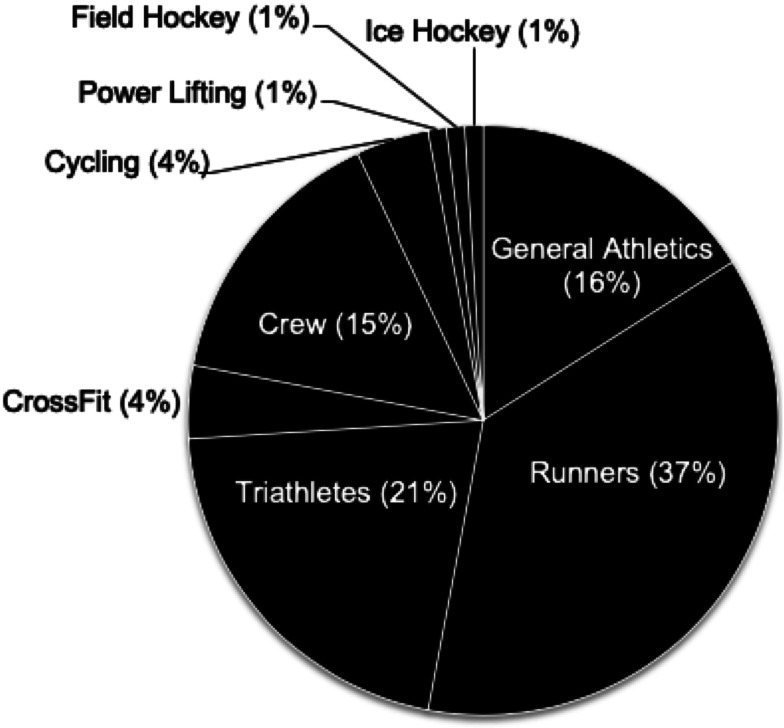
Sport classification of Masters Athletes.

Specific athletic activities reported by participants are included in Fig. 1.

The average energy intake for the total sample was 7995⋅3 ± 3461⋅8 kilojoules (kJ)/d [1910 ± 827 kilocalories (kcal)].

### Dietary protein intake and the US RDA

All dietary results are presented in Table 2. The average percent energy intake from protein was 15⋅6 ± 2⋅6 %, which is consistent with the 10–35 % recommended by the Acceptable Macronutrient Distribution Ranges (AMDR)^(16)^. The average protein intake was 1⋅43 ± 0⋅53 g/kg LBM for the total sample. No differences existed between women (1⋅49 ± 0⋅53) and men (1⋅36 ± 0⋅53) in g/kg LBM, (*t*(174) = −1⋅55, *P =* 0⋅12). When compared to the US RDA using total body weight, all participants, on average, consumed 1⋅0 ± 0⋅4 g/kg total body weight of dietary protein. The total sample had higher protein intakes compared to the US RDA using LBM as a reference, (*t*(175) = 15⋅73, *P* < 0⋅001). Both women (*t*(93) = 12⋅69, *P* < 0⋅001) and men (*t*(81) = 9⋅56, *P* < 0⋅001) independently had higher protein intakes (g/kg LBM) compared to the US RDA.

**Table 2.** Energy and protein intakes for the total sample and for women and Men

	Total Sample (*n* 176)	Standard Deviation	Women (*n* 94)	Standard Deviation	Men (*n* 82)	Standard Deviation	*P* value
Protein intake							
g/d	73⋅2	30.4	64⋅2*	20.9*	83⋅6	36.9	<0⋅001
g/kg total body weight	1⋅0	0.4	1⋅0	0.38	1⋅0	0.45	0⋅456
g/kg lean body mass	1⋅43	0.53	1⋅49 ± 0⋅53	0.53	1⋅36	0.53	0⋅124
Energy intake							
kJ/d (kcal/d)	7995⋅3 (1,910⋅4 )	3461.8 kJ/d 1910.4 kcal/d	6966⋅8* (1664⋅3*)	2372.2* kJ/d 566.7* kcal/d	2191⋅8 (2191⋅8 )	4094.7 kJ/d 978.2 kcal/d	<0⋅001
kJ/kg total body weight (kcal/kg total body weight)	111⋅8 (26⋅7 )	47.7 kJ/kg 11.4 kcal/kg	108⋅8 (26⋅0)	42.7 kJ/kg 10.2 kcal/kg	115⋅5 (27⋅6	52.7 kJ/kg 12.6 kcal/kg	0⋅353
kJ/kg lean body mass (kcal/kg lean body mass)	156⋅1 (37⋅3 )	61.1 kJ/kg 14.6 kcal/kg	161⋅6 (38⋅6)	59.4 kJ/kg 14.2 kcal/kg	150⋅3 (35⋅9 )	62.4 kJ/kg 14.9 kcal/kg	0⋅227
Percent (%) of energy from protein	15⋅6	2.6	15⋅7	2.6	15⋅4	2.6	0⋅588

g, grams; kg, kilograms; kJ, kilojoules; kcal, kilocalories

* Women's values are significantly lower than men's values.

## Discussion

The objective of the present study was to describe the average dietary protein intake in g/kg LBM and to explore the relationship between these two variables in a healthy sample. Our sample consumed 15⋅6 % of their energy as protein, well within the AMDR (10–35 %). Protein intake of 1⋅4 g/kg LBM was similar between women and men, with a protein-to-energy ratio of about 0⋅038 compared to the normative values for light and moderately active women (0⋅059 to 0⋅074) and men (0⋅081 to 0⋅098)^(17)^.

Based on previous research, it is clear that there are limitations when determining protein needs. As a reference standard, the US RDA for protein is established as a reference for consumers with respect to how much protein they should consume. These recommendations are designed to guide the general population in estimating protein needs, but have been critiqued for the accuracy across diverse populations. Notably, for active populations, it has been suggested that protein needs are closer to a range of 1⋅2–2⋅0 g/kg^(18)^. The US RDA for protein is a highly valuable tool for estimation; however, it may better translate as a minimum threshold for maintenance rather than a precise obtainable amount, which can have high individual variability. In support of this, it has been previously speculated that current protein recommendations based on the US RDA may be lower than physiological needs, especially in those who exercise regularly^(3,4,19)^. There are data suggesting that this technique tends to overestimate intake. Mitchell *et al.*^(20)^ conducted a 10-week randomised controlled trial where participants consumed protein at twice the US RDA. This increased protein intake resulted in a better retention of LBM in older adults (74⋅2 ± 3⋅6 years of age), suggesting that protein needs may be higher in older individuals, which is in agreement with others.^(21)^ In addition, protein intake within the AMDR of 10–35 % of energy intake routinely allows intake to exceed the US RDA^(22)^. Campbell *et al.*^(3)^ reported that meeting the US RDA for protein during a controlled 14-week eucaloric diet resulted in a significant loss of mid-thigh muscle area during a period with no weight loss in a group of healthy, women and men, 55–77 years of age. In an animal model, nitrogen balance data have displayed a significant underestimation of protein needs when compared against the requirements to maintain LBM measured by DXA^(23)^, which is consistent with our data. Furthermore, a biphasic linear regression analysis of the nitrogen balance data resulted in a higher value for protein recommendations (1 g/kg), which is consistent with our findings^(24)^. The critique of the current recommendations may be due to the failure of nitrogen balance data to account for other physiologic outcomes that are important to protein status. Therefore, the goal of our analyses was to describe protein intake using LBM, a primary physiological driver of protein needs, and compare these findings to the current recommendations.

The indicator amino acid oxidation (IAAO) technique is an alternative tool and has estimated adult protein needs to be around 1⋅2 g/kg^(24)^, which is in close agreement to the average intake we found. Like nitrogen balance, the IAAO technique uses the biological response under controlled conditions as an indirect estimation of a theoretical protein equilibrium. Nitrogen balance and the IAAO technique appear to be accurate measures of protein balance; however, they are not without their limitations, specifically, their inability to accurately account for body composition^(4,8,25)^.

In addition, the use of total body weight for the aforementioned methods leads to one of the biggest critiques of current protein recommendations. Total body weight is indicative of weight status, but does reflect body composition differences, which can lead to underestimation of protein needs. Notably, sarcopenia is a protein intake-related condition characterised by a reduced quantity of skeletal muscle mass, of which progression is directly measurable through body composition analysis^(26)^. Additionally, sarcopenic obesity is a new class of obesity in older adults in which low skeletal muscle mass is coupled with high levels of adiposity^(27)^. The lack of body composition data makes it extremely difficult to track the progression of conditions like sarcopenia and sarcopenic obesity.

In addition to body composition, methods like nitrogen balance and the IAAO do not account for physiological outcomes that are indicative of protein needs. Physiological outcomes are often measured as a sign of progression, related to protein-supported muscle hypertrophy, or regression related to protein-deficient muscle breakdown. LBM is a well-accepted outcome measure for protein status and can account for tissue distribution differences. Protein intakes have been positively associated with the development of LBM. In fact, increased protein intakes have been reported to improve LBM and functional capacity^(20,28–31)^. Higher intakes of protein also help to maintain LBM in hypocaloric weight loss diets^(32–34)^. In the elderly, loss of LBM was the lowest in those consuming the most protein (about 18 % of total energy intake)^(35)^.

As suggested, the US RDA for protein needs has been established using primarily nitrogen balance data. If nitrogen balance serves as a surrogate for LBM, then the influence of varying protein intakes following adaptation over time on LBM would be valuable. If the protein intakes required to maintain LBM were an outcome, it would be of great interest to appreciate the current protein intakes relative to LBM. Using maintenance of LBM as an outcome, instead of zero-nitrogen balance, may necessitate higher accuracy when estimating protein intake, especially in the elderly, those on bed rest, or during energy restriction. Limited data have been gathered to support LBM as a more accurate metric. The present study is in agreement with previous data exploring this relationship and suggests that there may be promise in using LBM to estimate protein needs^(4,8)^. Recently, Rafii *et al.*^(8)^ reported average protein needs of 1⋅62 ± 0⋅14 g/kg LBM based on the amino acid oxidation technique in a cohort of older women. These protein needs are slightly higher than the average protein intake consumed by our cohort^(8)^. In addition, we reported no significant differences in protein intake (g/kg LBM) between women and men. Women appeared to have slightly, but not significantly, higher protein intakes than men. This might be expected because women and men's protein intakes per gram of total mass are very similar (1⋅0 ± 0⋅38 *v.* 1⋅0 ± 0⋅45, respectively), and women typically have a lower proportion of lean mass compared to men^(36)^. We speculate that the lack of differences observed between women and men in protein intake (in g/kg LBM) may be due to indexing to LBM instead of total body weight, which is more relative to an individual's body composition. It is important to note that our analysis relies on the assumption that our healthy population was meeting their physiological protein needs. Based on the data presented here, it is still unclear whether the dosing of protein should be based on LBM, but our analysis is a start to how much protein it takes for healthy weight stable adults to maintain their LBM. Our overall findings may, therefore, contribute to the evolving perspective on how best to determine protein recommendations for healthy adults.

The present study was not without limitations, which included the use of self-reported dietary intake information. Self-reported data introduces the potential of self-reporting bias and/or the misrepresentation of information^(37)^. However, participants were not aware that protein was our nutrient of interest, therefore, reducing the potential to influence the results. Another limitation of the present study includes the lack of nitrogen balance data with which to corroborate the present results. Given the historic use of nitrogen balance in deriving the current US RDA, it would be beneficial to have had access to this information to better ascertain the present results. In addition, we maintained underlying assumptions: participants were weight and metabolically stable, and their intake was constant without yearly variation. To help account for limitations in weight stability, body weights were measured at two different sessions to compare weight discrepancies. The assumed weight stable status occurs without a change in body composition including LBM, or a change in protein turnover, without the need for habitual adaptation or accommodation. Stability, therefore, suggests a predictable interrelationship between LBM and protein intake. The limitations of relying on the assumptions of weight stability were uncontrolled in the present study and have the potential to negatively influence the data. With respect to potential variations in yearly protein intake, it is important to note that the FFQ attempts to account for this by collecting intake based on a yearly average. Finally, dietary measures of protein are absolute and do not take into account the quality of protein or the distribution of protein intake across the day. Protein quality can influence muscle protein synthesis, which can, in turn, alter LBM. In an effort to account for potential limitations in sample size, *post hoc* power analysis was applied with an effect size of 0⋅5 and with a sample size of 176, which resulted in a power of 0⋅99 for the total sample. This revealed an adequate sample size to detect differences from the US RDA. Despite the limitations, the data provide a valuable examination of the relationship between protein intakes in a cohort of healthy Masters Athletes.

In conclusion, in our cross-sectional analysis, we found that the average protein intake was significantly higher than the current US RDA in a cohort of healthy individuals. We also found a significantly higher protein need per kg LBM when compared to the US RDA for both women and men. Our intention is to establish the foreground for the relationship between LBM and protein need. In addition, we hope to establish the foundation for LBM to be used to calculate protein needs. Furthermore, there was no significant difference between women and men with respect to protein intake per kg of LBM. Therefore, LBM may be involved in the physiological driver of protein needs in physically active, healthy adults. Further exploration of the relationship between protein intake and LBM is warranted to support the notion of calculating protein needs based on LBM. These studies should consider longitudinal designs that monitor weight changes, energy and protein intakes in healthy as well as at-risk populations to better elucidate meeting physiological protein needs. In addition, nitrogen balance studies that monitor body composition should also be considered to better understand how lean mass reflects protein needs. By establishing the relationship, between protein need and LBM, we may increase our accuracy when developing protein recommendations for individuals, which, in turn, can increase the quality of care for all.
